# Antibody glyco-optimization and site-specific conjugation enhance the immune-stimulating activity of antibody–TLR ligand conjugates

**DOI:** 10.1016/j.jbc.2026.113269

**Published:** 2026-06-19

**Authors:** Xianyang Wang, Yaoxian Lou, Helena Yun, Margaryta Gomozkova, Guangming Li, Lishan Su, Lai-Xi Wang

**Affiliations:** 1 Department of Chemistry and Biochemistry, University of Maryland, College Park, Maryland, USA; 2 Division of Virology, Pathogenesis and Cancer, Department of Pharmacology and Physiology, Institute of Human Virology, University of Maryland School of Medicine, Baltimore, Maryland, USA

**Keywords:** antibody glycoengineering, Fc glyco-optimization, immune activation, immune-stimulating antibody conjugates (ISACs), site-specific bioconjugation

## Abstract

Immune-stimulating antibody conjugates (ISACs) represent an emerging class of immunotherapeutics that combine tumor-targeted antibodies with innate immune agonists. However, current ISAC platforms often rely on heterogeneous conjugation strategies and poorly defined antibody architectures, making it difficult to disentangle how Fc glycosylation, payload presentation, and linker design collectively govern immune activation. Here, we report a dual-enzymatic antibody-editing strategy that integrates Fc glyco-engineering with site-specific payload conjugation to construct structurally defined immune-stimulating antibody conjugates. Using microbial transglutaminase-mediated conjugation at the Fc Q295 residue together with Endo-S2-mediated Fc glycan remodeling at N297, we generated trastuzumab-based ISACs with defined Fc glycoforms and linker architectures. Systematic evaluation reveals that Fc glycosylation state and payload presentation act as orthogonal and synergistic determinants of immune activation. In particular, an intact and afucosylated Fc glycan is essential for robust tumor-cell killing and cytokine induction, while cleavable linkers further enhance innate immune activation through efficient intracellular release of the TLR7/8 agonist. This work establishes a versatile chemical strategy for controlling antibody architecture and immune activation, providing a broadly applicable platform for the rational design of next-generation antibody therapeutics.

Antibody–drug conjugates (ADCs) have transformed the landscape of targeted cancer therapy by taking advantage of the tumor specificity of monoclonal antibodies to deliver highly toxic payloads to target cells. Clinically approved ADCs have primarily employed microtubule inhibitors or DNA-damaging agents as payloads, exploiting their sub-nanomolar potency to induce direct tumor cell death (1, 2, 3, 4). While these agents have demonstrated impressive clinical benefit, their mechanism of action is largely restricted to direct cytotoxicity from the released payloads (5, 6).

To address these limitations, recent efforts have expanded the ADC concept to incorporate immunostimulatory agents capable of eliciting durable antitumor immunity (5, 6, 7, 8). Among these, immune-stimulating antibody conjugates (ISACs) are designed to replace the cytotoxic payload of conventional ADCs with agonists of pattern recognition receptors (PRRs), a family of innate immune sensors, including Toll-like receptors (TLRs), C-type lectin receptors, NOD-like receptors, and RIG-I-like receptors (9, 10, 11, 12, 13, 14, 15, 16, 17). Engagement of PRRs on dendritic cells, macrophages, and other myeloid populations triggers proinflammatory cytokine release, enhances antigen presentation, and facilitates the priming and recruitment of tumor-specific T cells. This dual mode of action, consisting of antigen-targeted delivery combined with localized immune activation, offers the potential to convert “cold” tumors into “hot” tumors, thereby sensitizing them to immunotherapy (18). Ackerman and co-workers have recently demonstrated that conjugation of the TLR7/8 agonist, T785, to tumor-specific antibodies can potently activate tumor-infiltrating myeloid cells and promote antitumor T cell responses *in vivo*, providing proof-of-concept for ISAC-mediated immune activation (19). Indeed, several ISAC candidates have advanced into early-stage clinical evaluation, demonstrating the ability to promote localized immune activation within tumors while reducing the systemic toxicity associated with free PRR agonists. Despite this initial clinical progress, however, most first-generation ISAC programs have encountered substantial limitations that have curtailed their therapeutic potential. Clinical studies have revealed recurrent challenges related to immunogenicity, pharmacokinetics, and tolerability. For example, the HER2-targeted TLR7 agonist ISAC NJH395 has elicited anti-drug antibodies in all treated patients and showed only modest efficacy at tolerated doses, ultimately leading to program discontinuation. Similarly, a HER2-directed TLR7/8 ISAC (BDC-1001) was halted due to unfavorable pharmacokinetic properties that severely limited effective tumor exposure, while a TLR8 agonist-antibody conjugate (SBT-6050) was discontinued following the emergence of significant immune-related toxicities. Collectively, these outcomes indicate that although the ISAC concept is clinically promising, current implementations remain far from optimal (20, 21, 22, 23).

At a mechanistic level, these limitations largely stem from the design principles of existing ISAC platforms. Most reported ISACs used nonspecific conjugation strategies, resulting in heterogeneous drug–antibody ratios (DARs) and poorly defined payload positioning, factors that can adversely impact pharmacokinetics, tolerability, and efficacy. In addition, the interactions between the Fc domain and Fcγ receptors are critical for recruitment of effector cells and for antibody internalization, and these interactions are strongly influenced by Fc glycosylation patterns (24, 25, 26). Yet, Fc glycan composition has largely been overlooked in current ISAC designs (Fig. 1). In this context, there is a clear need for next-generation ISAC platforms that integrate site-specific conjugation, precisely controlled DAR, and rational Fc glyco-engineering to maximize immune activation while minimizing off-target effects. Here we present a dual enzymatic site-specific conjugation strategy for constructing structurally well-defined ISACs, which combines microbial transglutaminase (mTG)-mediated antibody conjugation at the Q295 site (27) with a specific TLR agonist (T785) and the endoglycosidase S2 mutant (Endo-S2 mutant)-mediated glycoengineering to optimize the Fc glycans at the N297-site for high affinity binding to FcγRs (28, 29). We found that this orthogonal strategy generated structurally homogeneous, Fc glycosylation-optimized ISACs with markedly enhanced FcγIIIa receptor binding affinity and a well-defined drug-to-antibody ratio (DAR). Studies with human tumor and immune cells showed that an intact N297 glycan was indispensable for the ISAC activity, and that afucosylated Fc glycoforms further augment FcγRIIIa engagement, tumor-cell killing, and cytokine induction. Collectively, these findings highlight the power of integrating precise conjugation chemistry with Fc-glycosylation optimization to produce potent immune-stimulating antibody conjugates.

**Figure 1:**
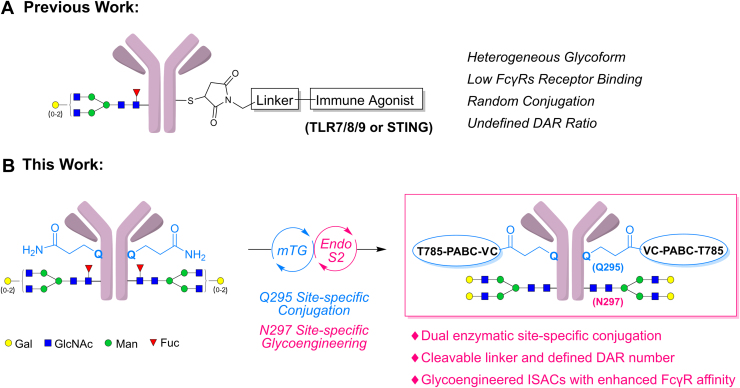
**Dual enzymatic site-specific strategy for homogeneous ISAC construction.***A*, conventional ISAC design resulting in heterogeneous conjugation, variable DAR, and limited control of payload positioning and Fc glycosylation. *B*, dual-enzymatic strategy integrating site-specific conjugation at Q295 with Fc glycan remodeling at N297 to generate structurally defined ISACs with controlled DAR and optimized Fc function.

## Results and discussion

### Design of the target homogeneous ISACs

To construct structurally well-defined ISACs and enable systematic evaluation of the roles of Fc glycosylation and payload presentation in immune activation, we designed a dual-enzymatic strategy integrating site-specific TLR ligand conjugation with Fc glyco-optimization to generate ISACs with precise and homogeneous architectures (Fig. 1). Trastuzumab, an anti-HER2 therapeutic monoclonal antibody, was chosen as the antibody to demonstrate the concept. We sought to introduce a small-molecule TLR agonist (T785) at the Fc glutamine-295 (Q295) site by microbial transglutaminase (mTG) and to optimize the Fc glycosylation for high-affinity binding to Fcγ receptors by endoglycosidase-based Fc glycan remodeling. mTG has been shown to be able to perform trans-glutamination specifically at Q295 upon Fc deglycosylation with PNGase F (27, 30, 31). Although an mTG mutant with improved activity toward native antibodies has been described, efficient conjugation typically requires large amounts of enzyme and extended incubation times (32). More recently, Zhou and co-workers have shown that mTG can efficiently transfer a tag or a fluorescent probe at the Q295 residue when the antibody is deglycosylated with an endoglycosidase (Endo-S2), leaving the innermost GlcNAc moiety of the Fc glycan remaining at the glycosylation site (33). We reasoned that the GlcNAc moiety provides a handle for subsequent enzymatic remodeling to optimize the Fc glycosylation, using an endoglycosynthase (Endo-S2 D184M mutant) as the enzyme, to introduce an afucosylated fully galactosylated complex type N-glycan, which shows high affinity for FcγRIIIa (28, 29). In this context, it will be interesting to evaluate how efficiently the enzymatic Fc glycan remodeling will work when the Q295 site is occupied by a TLR ligand. In addition, to enable intracellular release of the TLR agonist following antibody internalization, we incorporated a cleavable valine-citrulline-*p*-aminobenzylcarbamate (VC-PABC) linker into the antibody-ligand conjugates. The Val-Cit dipeptide can be selectively cleaved by lysosomal proteases such as cathepsin B, triggering fragmentation of the PABC spacer and release of the active TLR agonist.

### Synthesis of the toll-like receptor seven-eighths agonist (T785)-cleavable linker conjugate

**Figure 2:**
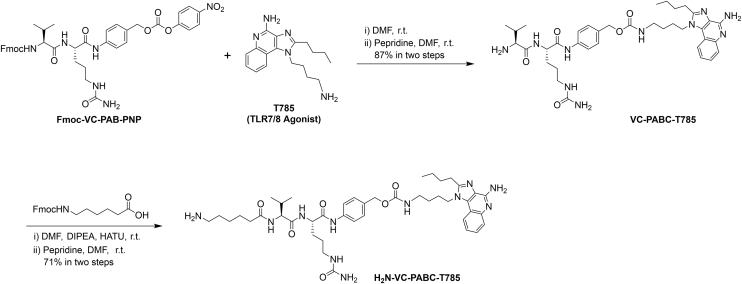
**Synthesis of the TLR ligand conjugated with a cleavable linker (H_2_N-VC-PABC-T785)**.

To introduce a valine-citrulline-*p*-aminobenzylcarbamate (VC-PABC) linker to the toll-like receptor seven-eighths agonist (19), T785, the *p*-nitrophenol ester-activated VC dipeptide-PAB derivative (Fmoc-VC-PAB-PNP) was coupled with T785. Subsequent removal of the Fmoc protecting group afforded the linker-payload conjugate VC-PABC-T785. To facilitate the mTG-catalyzed transglutamination reaction, a spacer was introduced by coupling the intermediate with N-Fmoc-6-aminohexanoic acid, followed by Fmoc deprotection to yield the amine-functionalized derivative H_2_N-VC-PABC-T785. This compound serves as the donor substrate for the subsequent mTG-catalyzed transglutamination reaction (Fig 2).

### Synthesis of the immune-stimulating antibody conjugates

**Figure 3:**
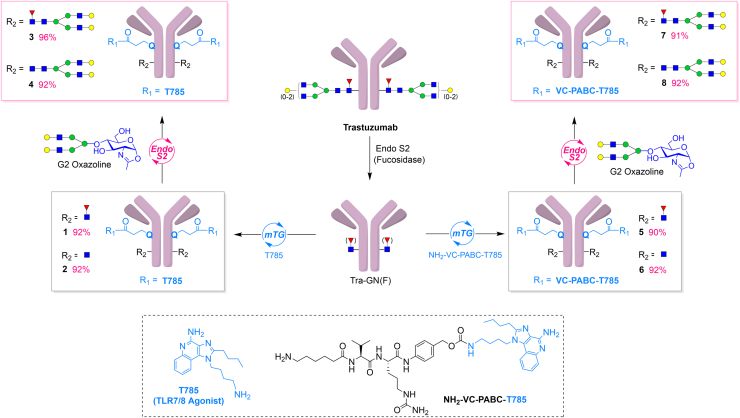
**Synthesis of the immune-stimulating antibody conjugates (ISACs 1–8)**.

**Figure 4:**
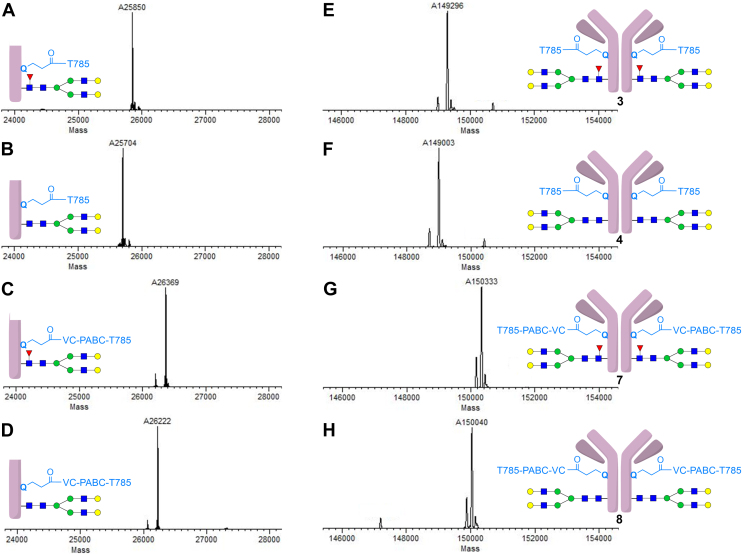
**LC-MS characterization of site-specifically conjugated ISACs.***A–D*, deconvoluted MS spectra of IdeS-digested Fc fragments from ISACs 3 to 4, and 7 to 8. *E–H*, intact-antibody MS spectra of the corresponding ISAC conjugates.

To integrate these complementary catalytic functions, we designed a modular workflow in which mTG-mediated Q295 conjugation and Endo-S2-based N297 glycan remodeling operate sequentially to yield structurally homogeneous, Fc glycosylation-optimized ISACs (Fig. 3). In the first step, Trastuzumab was subjected to deglycosylation using immobilized Endo-S2 to generate the core-fucosylated glycoform (Tra-GNF) (34). Employing the α1,6-fucosidase from *Lactobacillus casei* (AlfC) previously reported by our group (34), the α1,6-defucosylation was rapidly and efficiently completed, yielding the defucosylated antibody intermediate (Tra-GN). These deglycosylated antibodies were then used as substrates for mTG-mediated site-specific conjugation at Q295 (Fig. 4). Two TLR7/8 agonist derivatives were used: T785 with a cleavable linker (NH_2_-VC-PABC-T785) and T785 without a cleavable linker (T785). The reactions afforded the antibody conjugates **1** (GNF-T785) and **2** (GN-T785) without a cleavable linker in the conjugates, as well as **5** (GNF-VC-PABC-T785) and **6** (GN-VC-PABC-T785), where the antibody conjugates carry a cleavable linker. The antibody-conjugates have a defined DAR of 2 and were isolated in excellent yields (90–92%). The identity and homogeneity of the antibody-conjugates were confirmed by LC-MS analysis (Supporting Information, Figs. S3–S6).

In the second enzymatic step, the truncated GlcNAc moiety at N297 was remodeled by Endo-S2 D184M-mediated transglycosylation using synthetic G2 glycan oxazoline. We found that the pre-installation of the TLR agonist (T785) at the Q295 site proximal to the N297 glycosylation site did not significantly affect the efficiency of Endo-S2 mutant (28) for transferring the glycans from the glycan oxazoline substrate. Thus, the Endo-S2 catalyzed glycosylation of the deglycosylated antibodies (**1**, **2**, **5**, and **6**) gave the corresponding glycosylated antibody conjugates in high yields (91–96%). These include ISAC **3** (G2F-T785) and ISAC **4** (G2-T785) without a cleavable linker, as well as ISAC **7** (G2F-VC-PABC-T785) and ISAC **8** (G2-VC- PABC-T785) with a cleavable linker (Fig.3). In addition, antibody conjugates **4** and **8** carry an afucosylated G2 glycan that are expected to have high affinity for FcγIIIA receptor, whereas conjugates **3** and **7** carry a core-fucosylated Fc glycan that are expected to have relatively low affinity for FcγIIIA receptor (29).

LC-MS analysis of the final ISACs confirmed precise and homogeneous modification. The IdeS-digested Fc fragments (Fig. 4, *A*–*D* and S7–S10) verified that both the T785 payload and G2 glycans were installed at the designated Fc sites, while the intact-antibody spectra (Fig. 4, *E*–*H* and S7–S10) showed single, well-defined molecular species with the expected mass shifts, confirming structural homogeneity and DAR = 2. SDS-PAGE analysis further supported the purity and integrity of the modified ISACs (Fig. S11). This modular, dual-enzymatic workflow thus enables complete control over both payload attachment and Fc glycan composition, providing a robust platform for systematically probing the contribution of Fc glycoforms to ISAC potency.

### Binding of the synthetic immune-stimulating antibody conjugates with FcγRIIIa and HER2-positive SKBR3 cells

**Figure 5:**
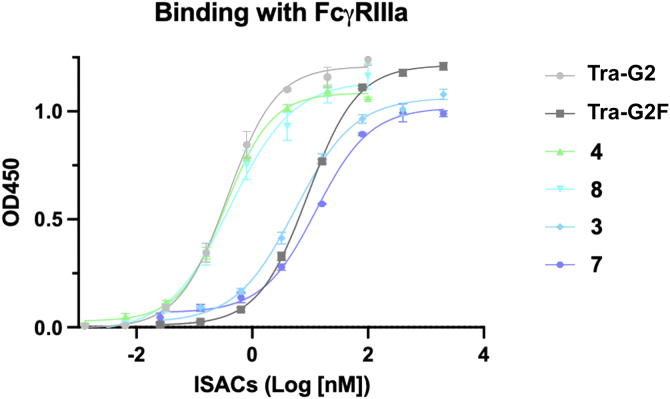
**Binding of ISACs to FcγRIIIa. FcγRIIIa-V158 (0.5 μg/ml) was coated onto a high-binding 96-well plate overnight at 4 °C**. For afucosylated ISACs (4 and 8), concentrations ranged from 100 nM to 0.00128 nM (fivefold serial dilutions); for fucosylated ISACs (3 and 7), from 2000 nM to 0.0256 nM (fivefold serial dilutions).

With these homogeneous ISACs in hand, we next evaluated how the Q295-specific TLR7/8 conjugation influences antibody’s affinity for FcγRIIIa binding by ELISA analysis. As shown in Figure 5, all ISACs (3–4, 7–8) displayed FcγRIIIa binding profiles comparable to their corresponding unconjugated antibodies (Tra-G2, Tra-G2F), indicating that site-specific modification at Q295 preserves Fc conformation and receptor recognition. Consistent with the well-established role of afucosylation in enhancing Fc-FcγRIIIa interactions (35), G2-bearing ISACs (4, 8) and Tra-G2 exhibited markedly higher binding affinity than their fucosylated counterparts (3, 7 and Tra-G2F), regardless of linker type. In addition, flow cytometry analysis using HER2-positive SKBR3 cells showed that the dual-modified ISACs retained HER2-binding capability comparable to that of unmodified trastuzumab at both 5 nM and 100 nM (Figs. S12–S13), suggesting that Q295 conjugation and Fc glycan remodeling did not measurably impair antigen recognition on the cancer cell surface.

### Depletion of HER2+ cancer cells by the antibody conjugates in the presence of human PBMCs

**Figure 6:**
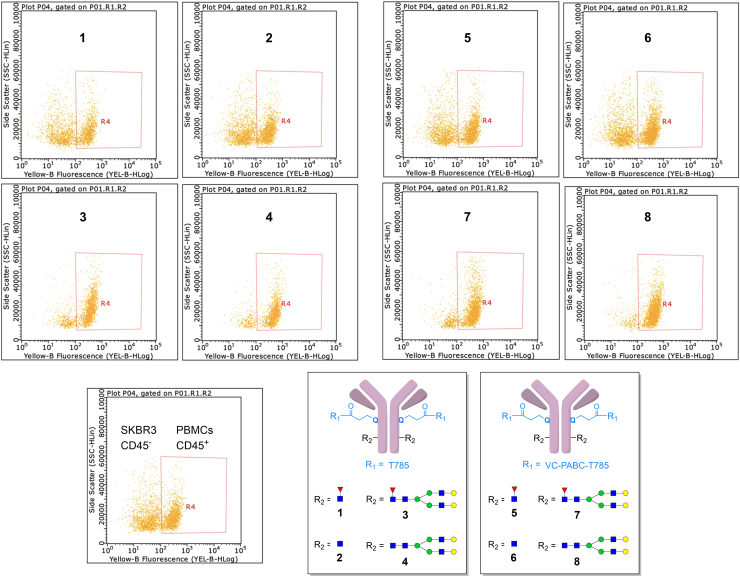
**Representative flow cytometry plots showingSKBR3 depletion in co-cultures with PBMCs after 96 h treatment with ISACs (5 nM).** Human PBMCs and SKBR3 breast cancer cells were co-cultured at a PBMC:SKBR3 ratio of 5:1 (1 × 10^5^ PBMCs and 2 × 10^4^ SKBR3 cells per well) in RPMI 1640 medium. After 96 h incubation at 37 °C with 5% CO_2_, cells were harvested and stained with anti-CD45-PE and 7-AAD to distinguish CD45^+^ PBMCs from CD45^-^ tumor cells.

Having confirmed that Fc structural integrity was preserved, we next examined whether these molecular differences translate into distinct biological outcomes. We assessed the tumor-killing potential of the engineered ISACs in a co-culture assay using HER2+ SKBR3 breast cancer cells and human peripheral blood mononuclear cells (PBMCs). SKBR3 and PBMCs were mixed at a 1:5 ratio and treated with 5 nM or 100 nM of each ISAC variant for 96 h. Flow cytometry analysis (Fig. 6) revealed that constructs 3 to 4, 7 to 8 bearing further elaborated biantennary N-glycans (G2F or G2) triggered marked depletion of tumor cells, whereas glycan-truncated variants 1 to 2, 5 to 6 (GNF and GN) elicited only marginal cytotoxicity irrespective of the linker type. These results suggest that a structurally intact N297 glycan is essential for productive FcγR engagement and subsequent effector-mediated tumor killing.

**Figure 7:**
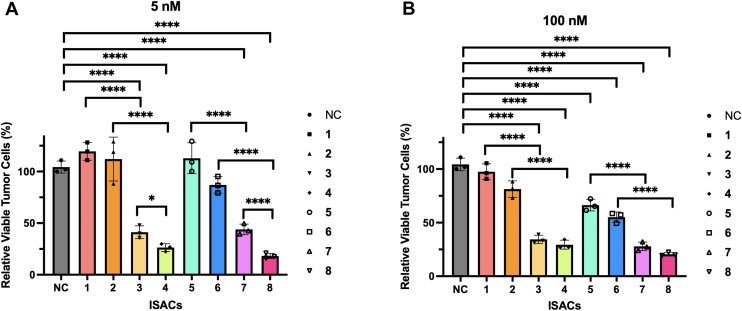
**Quantitative analysis of SKBR3 killing in SKBR3-PBMC co-cultures treated for 96 h with ISACs 1 to 8 at (*A*) 5 nM or (*B*) 100 nM.** Data represent mean ± SEM from three independent experiments (N = 3). Error bars indicate SEM. Statistical significance was determined by two-way ANOVA; ∗*p* < 0.01, ∗∗∗*p* < 0.001, ∗∗∗∗*p* < 0.0001.

Quantitative analysis of viable tumor cells (Fig. 7) further highlighted the potency advantage conferred by afucosylated Fc glycans. At 5 nM, G2 ISACs (4 and 8) achieved significantly greater tumor cell depletion than their G2F counterparts (3 and 7), whereas at 100 nM, both afucosylated and fucosylated G2 constructs reached comparable maximal efficacy. This concentration-dependent potency gap suggests that afucosylation enhances FcγRIIIa-mediated effector function, enabling strong cytotoxic activity at relatively low doses. These findings confirm that full elaboration of the N297 glycan is indispensable for ISAC-mediated killing, while removal of core fucose further augments the anticancer potency, particularly under sub-saturating conditions.

### Cytokine induction by engineered ISACs

**Figure 8:**
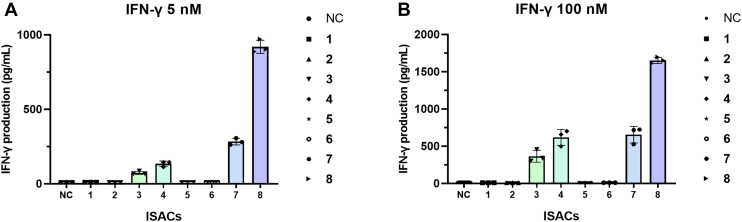
**IFN-γ secretion induced by ISAC variants in SKBR3/PBMC co-cultures.** Human PBMCs were co-cultured with SKBR3 breast cancer cells at a PBMC:SKBR3 ratio of 5:1 in RPMI 1640 medium. ISACs were added at final concentrations of 5 nM (*A*) or 100 nM (*B*), and culture supernatants were collected after 24 h. Data represent mean ± SEM from three independent experiments (N = 3). Error bars indicate SEM.

We also analyzed the cytokine release stimulated by the ISAC constructs. Consistent with tumor killing activity, IFN-γ secretion measured from co-culture supernatants at 24 h revealed a clear dependence of cytokine induction on the presence and structure of the Fc glycoforms (Fig. 8). Afucosylated G2 ISACs (4, 8) elicited the strongest IFN-γ responses, while their fucosylated G2F counterparts (3, 7) showed moderate induction. In striking contrast, ISACs lacking an N297 glycan (GNF and GN; 1, 2, 5, 6) failed to promote any significant cytokine release, confirming that full glycan elaboration is indispensable for FcγR engagement, downstream immune activation, and tumor cell killing. Notably, within a given glycoform, cleavable T785 linkers conferred a substantial advantage: constructs with a VC-PABC linker (8 in the G2 series, 7 in the G2F series) stimulated much higher IFN-γ production than the corresponding non-cleavable counterparts (4 and 3, respectively). This enhanced cytokine induction likely stems from more efficient intracellular payload release, as cleavable linkers such as Val-Cit are selectively processed by lysosomal proteinase (cathepsins) to liberate free T785 agonists that engage endosomal TLR7/8 receptors. In contrast, non-cleavable linkers require complete proteolytic degradation of the antibody scaffold, potentially resulting in slower and less efficient TLR activation, and may yield antibody fragment-payload complexes with suboptimal receptor accessibility or diminished agonistic activity compared to fully released small molecule ligands. Taken together, these results demonstrate that both Fc glycoform optimization and cleavable linker design are pivotal for maximizing ISAC immunostimulatory efficacy.

## Conclusion

This study establishes a modular chemoenzymatic strategy for constructing structurally defined and homogeneous immune-stimulating antibody conjugates (ISACs). By orthogonally integrating site-specific transglutaminase-mediated conjugation with Endo-S2-enabled Fc glycan remodeling, this platform enables precise and independent control of payload presentation and Fc glycoform composition within a single antibody scaffold. Cell-based immune activation studies further reveal that optimized Fc glycosylation and cleavable linker-mediated payload release act as synergistic determinants of maximal immune stimulation. More broadly, this dual-enzymatic strategy establishes a general antibody editing platform that integrates Fc glyco-engineering with site-specific conjugation for the rational design of antibody-based therapeutics.

## Experimental procedures

### Preparation of trastuzumab

Trastuzumab was recombinantly expressed in HEK293T cells and purified in-house by Protein A affinity chromatography, following our previously reported procedures (36).

### Preparation of ISACs 1 to 2 and 5 to 6 by mTG-catalyzed site-specific conjugation

A solution of Trastuzumab-GNF (2.5 mg) or Trastuzumab-GN (2.5 mg) in phosphate buffer (100 mM, pH 7.0; final antibody concentration 20 mg/ml) was incubated with microbial mTG (final enzyme concentration 0.5 mg/ml) in the presence of either T785 or VC-PABC-T785 (90 equiv) at 30 °C for 2 to 4 h. LC-MS analyses indicated complete conjugation at Q295 (Figs. S3–S6). The mixture was purified by Protein A chromatography and exchanged into PBS buffer (100 mM, pH 7.4) to yield the homogeneous ISACs 1 to 2 and 5 to 6. Compound 1 (2.30 mg, 92%): ESI-MS, calcd for the Fc domain released by IdeS treatment, M = 24,427 Da; found, 24,429 Da (deconvolution data); calcd for intact antibody, M = 146,454 Da; found, 146,454 Da (deconvolution data). Compound 2 (2.30 mg, 92%): ESI-MS calcd for the Fc domain released by IdeS treatment, M = 24,281 Da; found, 24,281 Da (deconvolution data); calcd for intact antibody, M = 146,159 Da; found, 146,162 Da (deconvolution data). Compound 5 (2.25 mg, 90%): ESI-MS, calcd for the Fc domain released by IdeS treatment, M = 24,947 Da; found, 24,949 Da (deconvolution data); calcd for intact antibody, M = 147,491 Da; found, 147,492 Da (deconvolution data). Compound 6 (2.30 mg, 92%): ESI-MS, calcd for the Fc domain released from IdeS treatment, M = 24,799 Da; found, 24,801 Da (deconvolution data); calcd for intact antibody, M = 147,198 Da; found, 147,200 Da (deconvolution data).

### Preparation of ISACs 3 to 4 and 7 to 8 by Endo-S2 D184M-catalyzed transglycosylation

A solution of 1, 2, 5, or 6 (each 1.5 mg) in phosphate buffer (100 mM, pH 7.0; final antibody concentration 20 mg/ml) was incubated with Endo-S2 D184M (enzyme/substrate ratio = 1:200, w/w) in the presence of synthetic G2 oxazoline donor (40 equiv) at 30 °C for 30 to 45 min. LC-MS analyses indicated complete transglycosylation at N297 (Fig. 2, *A*–*H* and S7–S10). The reaction mixture was purified by Protein A chromatography and exchanged into PBS buffer (100 mM, pH 7.4) to yield the homogeneous ISACs 3 to 4 and 7 to 8. Compound 3 (1.44 mg, 96%): ESI-MS, calcd for the Fc domain released by IdeS treatment, M = 25,848 Da; found 25,850 Da (deconvolution data); calcd for intact antibody M = 149,294 Da; found 149,296 Da (deconvolution data). Compound 4 (1.38 mg, 92%): ESI-MS, calcd for the Fc domain released by IdeS treatment, M = 25,702 Da; found 25,704 Da (deconvolution data); calcd for intact antibody M = 148,999 Da; found 149,003 Da (deconvolution data). Compound 7 (1.36 mg, 91%): ESI-MS, calcd for the Fc domain released by IdeS treatment, M = 26,367 Da; found 26,369 Da (deconvolution data); calcd for intact antibody M = 150,331 Da; found 150,333 Da (deconvolution data). Compound 8 (1.38 mg, 92%): ESI-MS, calcd for the Fc domain released by IdeS treatment, M = 26,220 Da; found 26,222 Da (deconvolution data); calcd for intact antibody M = 150,038 Da; found 150,040 Da (deconvolution data).

### Binding of ISACs with FcγRIIIa (V158) by ELISA

FcγRIIIa (V158) (0.5 μg/ml, 100 μl/well) in PBS buffer (150 mM, pH 7.4) was coated onto a high-binding 96-well plate (Santa Cruz Biotechnology) at 4 °C overnight. After washing twice with PBS containing 0.05% Tween-20 (PBST, 200 μl/well), the plate was blocked with 1% bovine serum albumin (BSA) in PBS (200 μl/well) for 1 h at room temperature. Following two additional washes, serial dilutions of ISACs were added in PBST buffer (100 μl/well) and incubated for 1 h. For the afucosylated ISACs (4 and 8), the concentration range was 100-0.00128 nM (5-fold serial dilutions). For the fucosylated ISACs (3 and 7), the concentration range was 2000-0.0256 nM (5-fold serial dilutions). After five washes with PBST, the plate was incubated with HRP-conjugated anti-human IgG F(ab’)_2_ (0.16 μg/ml, 100 μl/well; Invitrogen) for 1 h. Following another wash, 3,3′,5,5′-tetramethylbenzidine (TMB) substrate (100 μl/well; Thermo Fisher Scientific) was added for signal development. The reaction was stopped with 2 N H_2_SO_4_ (100 μl/well), and absorbance at 450 nm was measured on a SpectraMax M5 microplate reader (Molecular Devices). Binding curves were generated using GraphPad Prism 10.

### HER2-binding analysis of ISACs on SKBR3 cells by flow cytometry

SKBR3 cells (ATCC HTB-30) were resuspended in McCoy’s 5a Medium (ATCC 30–2007) supplemented with 10% fetal bovine serum (FBS, non-heat-inactivated) and 1% (v/v) penicillin-streptomycin (100 U/ml penicillin and 100 μg/ml streptomycin), and aliquoted into a round-bottom 96-well plate, approximately 1 × 10^5^ cells/well. Add trastuzumab-WT or ISACs at final concentrations of 5 nM and 100 nM. Incubate for 30 min at 4 °C. Centrifuge at 2000 rpm for 5 min and remove the supernatant. Wash cells twice with cold PBS. Prepare staining mix in PBS buffer containing PE-conjugated goat anti-human IgG (H + L) (Thermo Fisher Scientific, Catalog PA1-86078) at the recommended dilution. Add 50 μl staining mix to each well, gently resuspend the cells, and incubate for 30 min at 4 °C in the dark. Add 200 μl PBS, centrifuge at 2000 rpm for 5 min, and discard the supernatant. Repeat the wash once. Resuspend cells in 200 μl 2% paraformaldehyde in PBS and store at 4 °C in the dark until flow cytometry analysis. Flow cytometric analysis was performed on Guava EasyCyte HT System (Millipore). Flow cytometry data and mean fluorescence intensity (MFI) values were analyzed with FlowJo software version 10.8.1 and plotted with GraphPad Prism 10.

### Co-culture cytotoxicity assay

Human buffy coats were obtained from the Gulf Coast Regional Blood Center, and peripheral blood mononuclear cells (PBMCs) were isolated by density gradient centrifugation using Ficoll-Paque PLUS (Cytiva) (37). Human PBMCs and SKBR3 breast cancer cells were co-cultured simultaneously in flat-bottom 96-well plates at a PBMC: SKBR3 ratio of 5:1 (1.0 × 10^5^ PBMCs and 2.0 × 10^4^ SKBR3 cells per well) in 200 μl of RPMI 1640 medium (Gibco) supplemented with 10% heat-inactivated FBS and 1% (v/v) penicillin-streptomycin (100 U/ml penicillin and 100 μg/ml streptomycin). ISACs were added at final concentrations of 5 nM or 100 nM, while wells without ISAC treatment served as negative controls. Co-cultures were incubated for 96 h at 37 °C with 5% CO_2_. Following incubation, cells were harvested and stained with anti-CD45-PE and 7-AAD (BioLegend) to distinguish PBMCs (CD45^+^) from SKBR3 tumor cells (CD45^-^). The proportion of viable tumor cells was quantified by Guava EasyCyt HT System (MD Millipore) and normalized to untreated controls. Data were analyzed using guavaSoft software and plotted with GraphPad Prism 10.

### Cytokine analysis

Supernatants from parallel co-cultures were collected after 24 h incubation and analyzed for IFN-γ secretion using a Human IFN-γ ELISA MAX Deluxe (BioLegend) following the manufacturer’s instructions. Absorbance at 450 nm was measured on the GloMax Explorer multimode microplate reader (Promega). Cytokine data were processed and visualized with GraphPad Prism 10.

## Data availability

All data are contained within the manuscript and supporting information.

## Supporting information

This article contains supporting information.

## Conflict of interest

The authors declare the following financial interests/personal relationships which may be considered as potential competing interests:

X. W., H. Y., and L. X. W. are inventors on a provisional patent application filed with the United States Patent and Trademark Office by University of Maryland College Park relevant to the work in this manuscript. All other authors have no competing interests.
