# Supplementary material for: Antibody glyco-optimization and site-specific conjugation enhance the immune-stimulating activity of antibody–TLR ligand conjugates
Source: J Biol Chem. 2026 Jun 19;302(8):113269. doi: 10.1016/j.jbc.2026.113269 (PMC13400241; doi:10.1016/j.jbc.2026.113269)
Supplement: Supplementary Material [file mmc1.pdf]

**Antibody glyco-optimization and site-specific conjugation enhance the immune-stimulating activity of antibody-TLR ligand conjugates**

Xianyang Wang[1], Yaoxian Lou[2], Helena Yun[1], Margaryta Gomozkova[1], Guangming Li[2], Lishan Su[2], Lai-Xi Wang[1,*]

[1] Department of Chemistry and Biochemistry, University of Maryland, College Park, Maryland 20742, USA

[2] Division of Virology, Pathogenesis and Cancer, Institute of Human Virology, Department of Pharmacology and Physiology, University of Maryland School of Medicine, Baltimore, Maryland 21201, United States.

*Corresponding Author. Email: wang518@umd.edu

**Table of Contents:**

General procedures……………………………………………………………S2

Chemical synthesis of $NH_2$-VC-PABC-T785 ………………………………...S3-S4

Fig. S3-S10. LC-MS profiles of the synthetic ISACs (**1**-**8**)…...……….……..S5-S12

Fig. S11. SDS-PAGE analysis of the ISACs..……………………….……..S13

Fig. S12. Flow cytometry analysis of HER2 binding by the ISACs..…...……..S14

Fig. S13. Quantification of HER2 binding by the ISACs..…..………………..S15

**General procedures**

Analytical reversed-phase HPLC was executed on a Thermo Scientific instrument with a YMC C18 column (5 μL, 4.6 × 250 mm). Preparative HPLC was performed on a Waters 600 HPLC instrument with preparative reverse-phase C18 column (Waters Symmetry 300, 19 × 300 mm) and dual UV detection at 210 and 254 nm. LC-MS analysis was performed on an Ultimate 3000 HPLC system coupled to an Exactive Plus Orbitrap mass spectrometer (Thermo Fischer Scientific) with C4 (whole antibody, gradient, 5-95% aq MeCN containing 0.1% FA for 6 min, 0.4 mL/min) C8 (IdeS digestion, gradient, 25-35% aq MeCN containing 0.1% FA for 6 min, 0.4 mL/min), or C18 (HRMS, gradient, 5-95% aq MeCN containing 0.1% FA for 6 min, 0.4 mL/min) column. Deconvolution data was transformed by MagTran software.

## Synthesis of $NH_2$-VC-PABC-T785

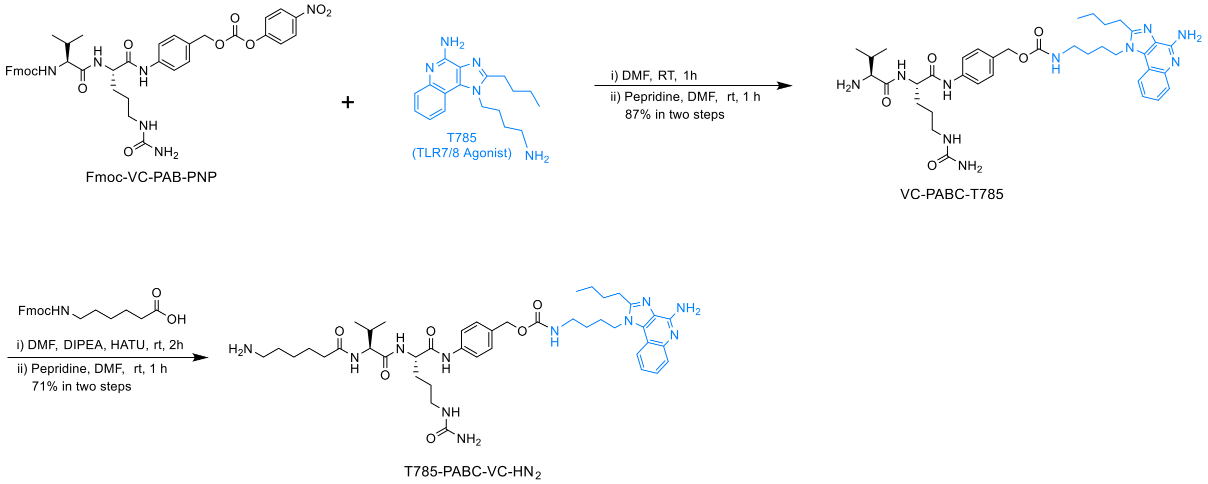


To a solution of Fmoc-VC-PAB-PNP (Ambeed) (50.0 mg, 0.065 mmol, 1.0 equiv) in anhydrous DMF (200 µL) was added T785 (Ambeed) (19.7 mg, 0.065 mmol, 1.0 equiv). The mixture was stirred at rt for 30 min. After the completion of the reaction as monitored by LC-MS, Piperidine (40 µL) was then added to remove the Fmoc group; after 30 min at rt, LC-MS indicated complete conversion to VC-PABC-T785. The crude was diluted with water (0.1% TFA) and purified directly by preparative HPLC (30-70% aq MeCN, 0.1% TFA, 40 min, 10 mL/min) to afford VC-PABC-T785 as a white powder (40.5 mg, 0.057 mmol, 87%). HRMS (ESI) m/z Calcd for $C_{37}H_{53}N_{15}O_5$ $[M+H]^+$, 717.4195, found, 717.4194.

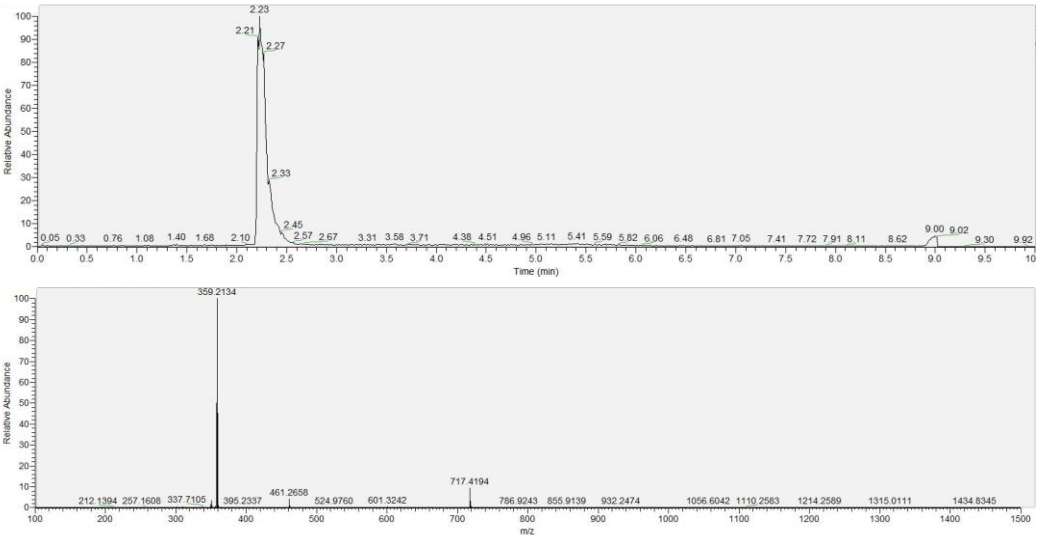


**Fig. S1.** HRMS analysis of the VC-PABC-T785

VC-PABC-T785 (25.0 mg, 0.035 mmol, 1 equiv) was dissolved in anhydrous DMF (400 μL), followed by sequential addition of Fmoc-6-aminohexanoic acid (12.2 mg, 0.035 mmol, 1 equiv), HATU (15.9 mg, 0.042 mmol, 1.2 equiv), and DIPEA (2.0 μL, 11.7 μmol, 6 equiv). The mixture was stirred at room temperature for 2 h, and reaction progress was monitored by LC-MS. Upon completion, the solvent was removed by lyophilization after addition of water (2 × 4.5 mL). The resulting residue was dissolved in 20% piperidine in DMF (v/v) and stirred at room temperature for 30 min to remove the Fmoc group. LC-MS analysis confirmed complete conversion to the desired $NH_2$-VC-PVBC-T785. The crude was diluted with water (0.1% TFA) and purified directly by preparative HPLC (25-65% aq MeCN, 0.1% TFA, 40 min, 10 mL/min) to afford $NH_2$-VC-PVBC-T785 as a white powder (20.7 mg, 0.025 mmol, 71%). HRMS (ESI) m/z Calcd for $C_{43}H_{64}N_{11}O_6$ $[M+H]^+$, 830.5036, found, 830.5041.

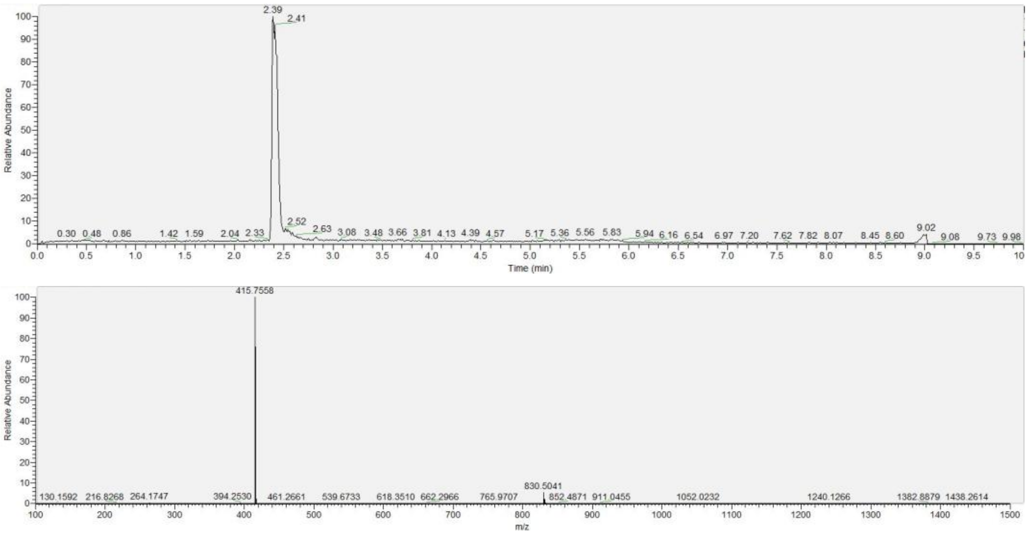


**Fig. S2.** HRMS analysis of the $NH_2$-VC-PVBC-T785

## LC-MS data of ISACs

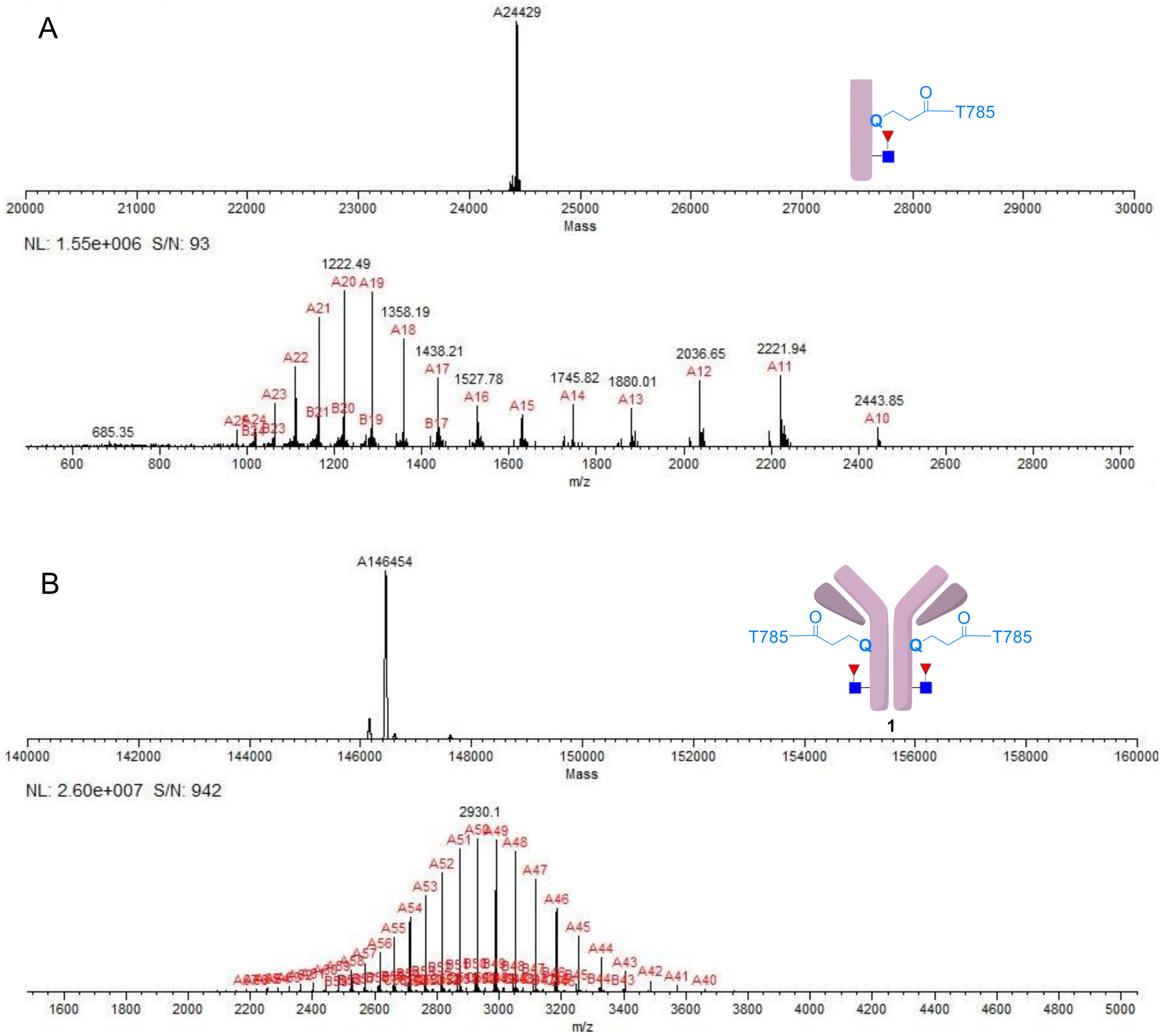


**Fig. S3.** A) Deconvoluted MS spectra of IdeS-digested Fc fragment from **1**. B) Intact-antibody MS spectra of **1**

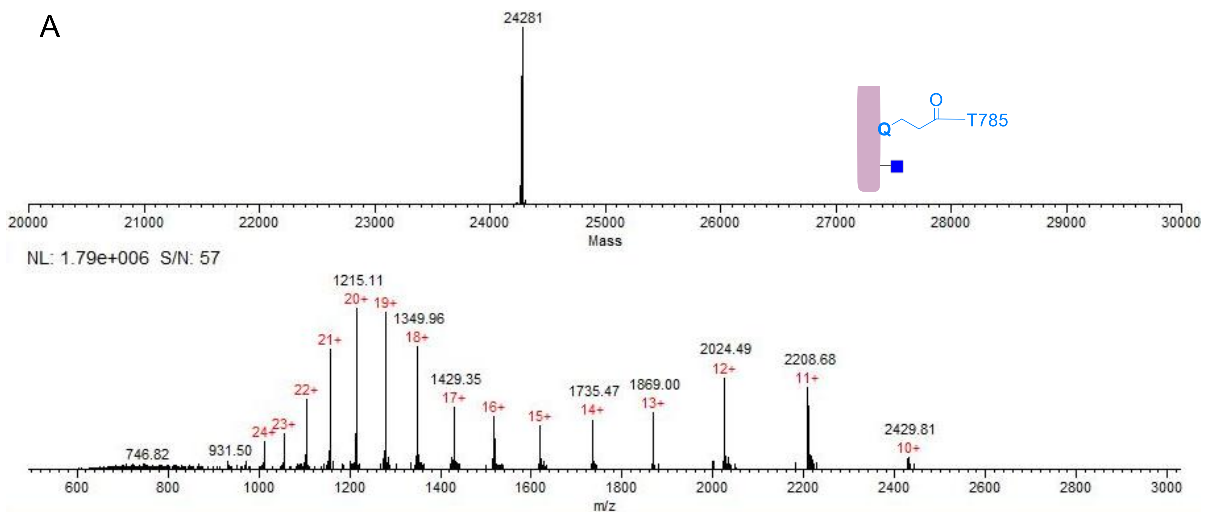


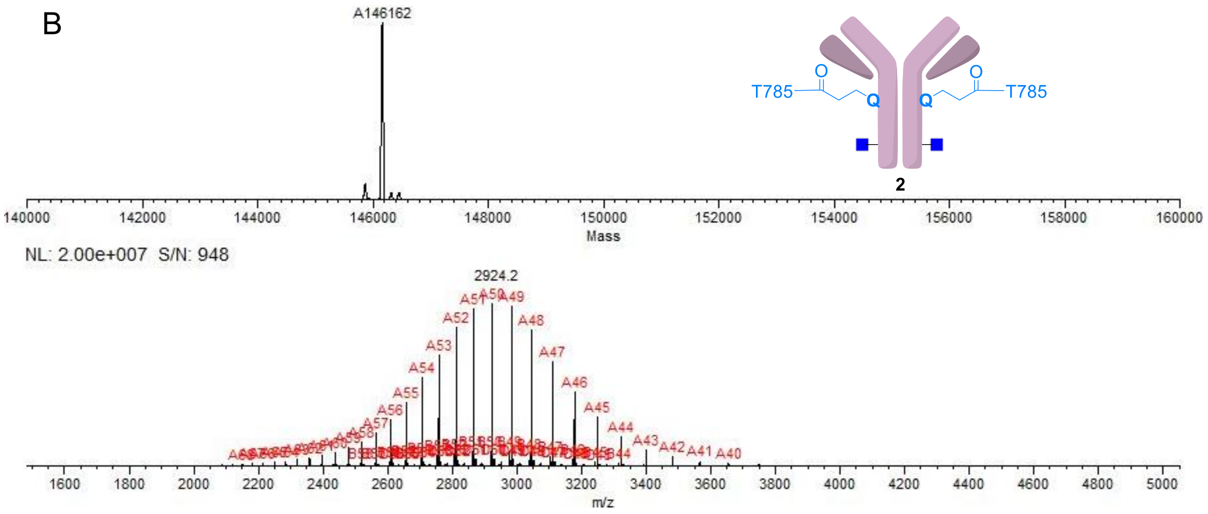


**Fig. S4.** A) Deconvoluted MS spectra of IdeS-digested Fc fragment from **2**. B) Intact-antibody MS spectra of **2**

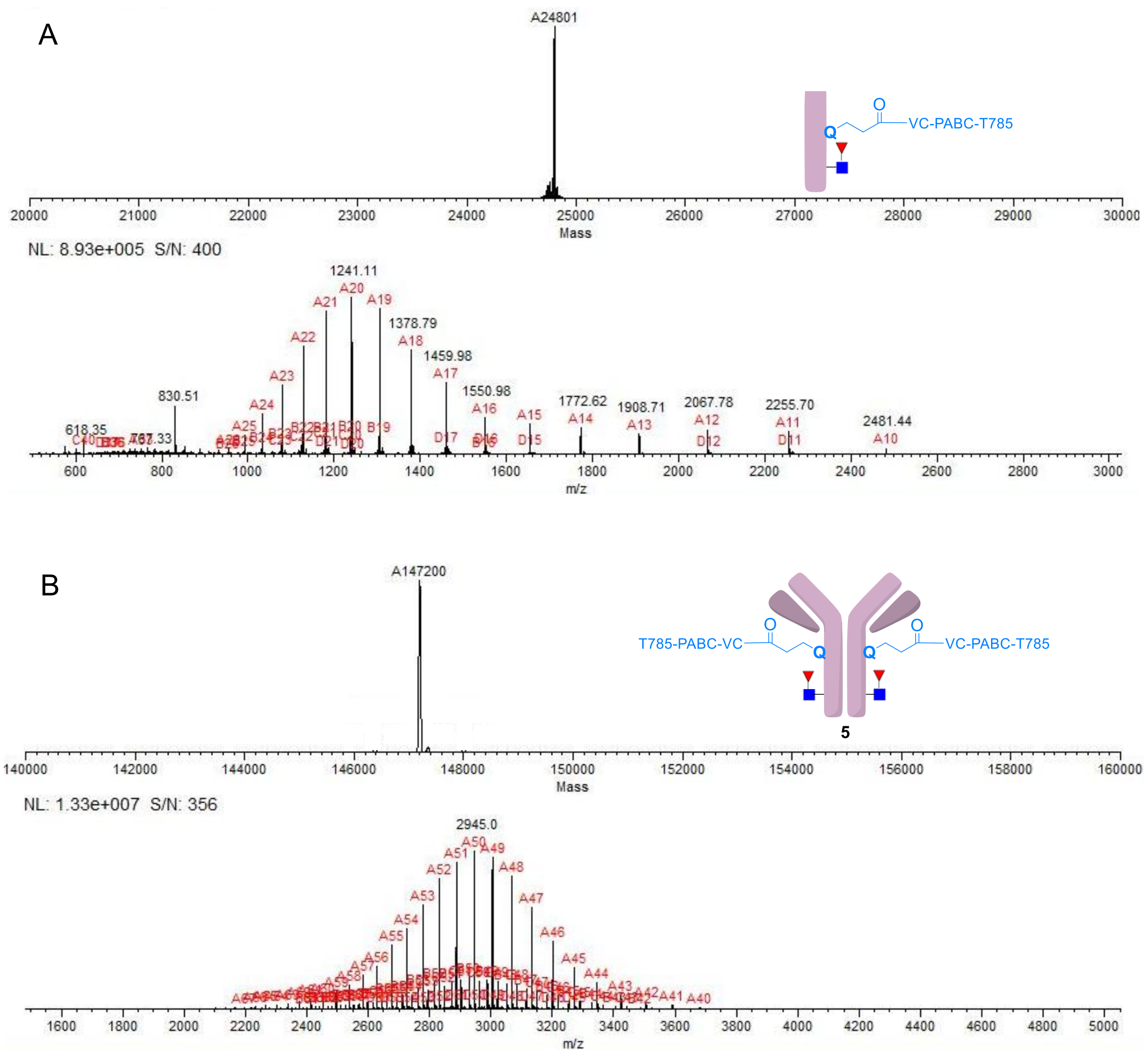


**Fig. S5.** A) Deconvoluted MS spectra of IdeS-digested Fc fragment from **5**. B) Intact-antibody MS spectra of **5**

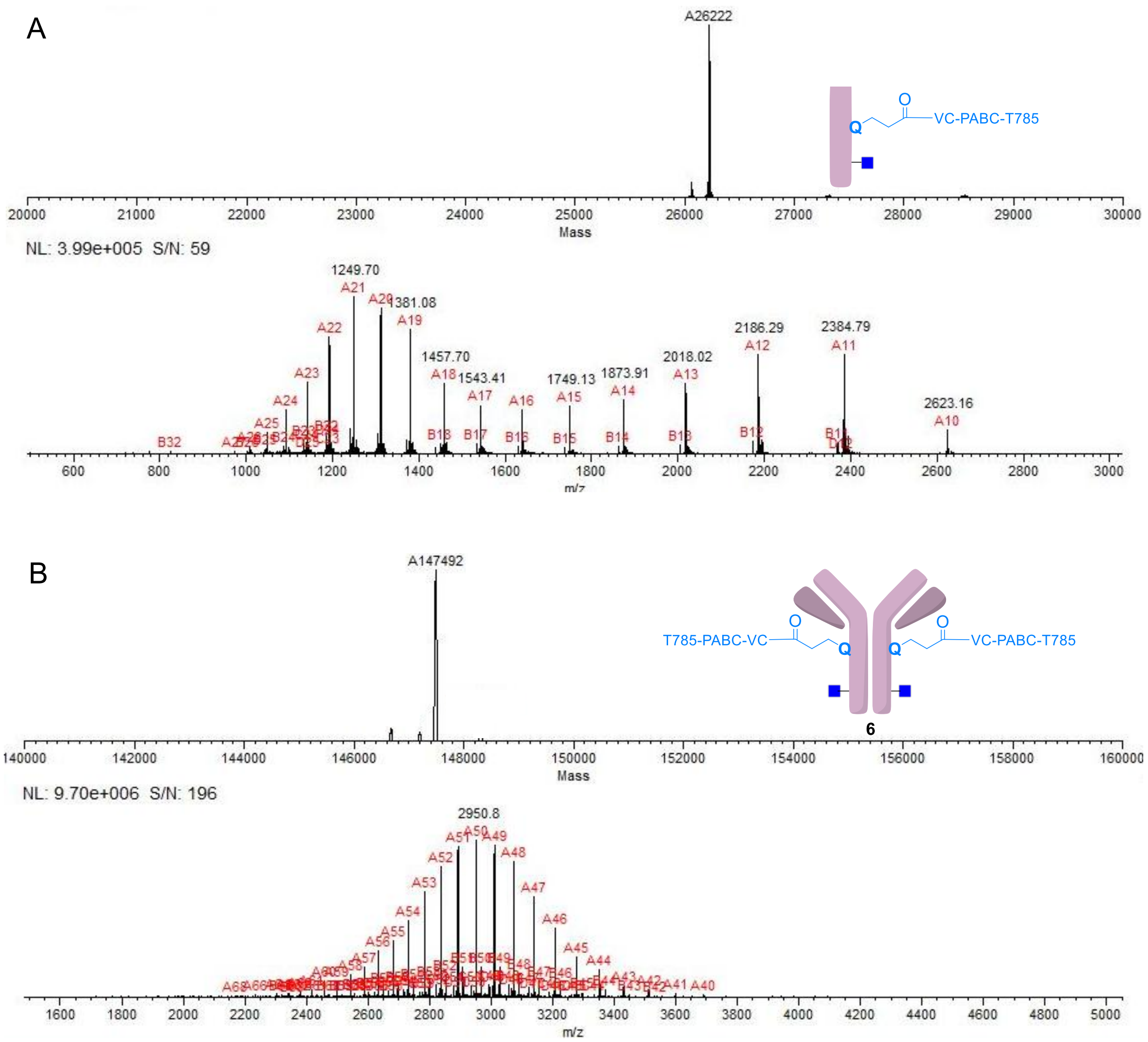


**Fig. S6.** A) Deconvoluted MS spectra of IdeS-digested Fc fragment from **6**. B) Intact-antibody MS spectra of **6**

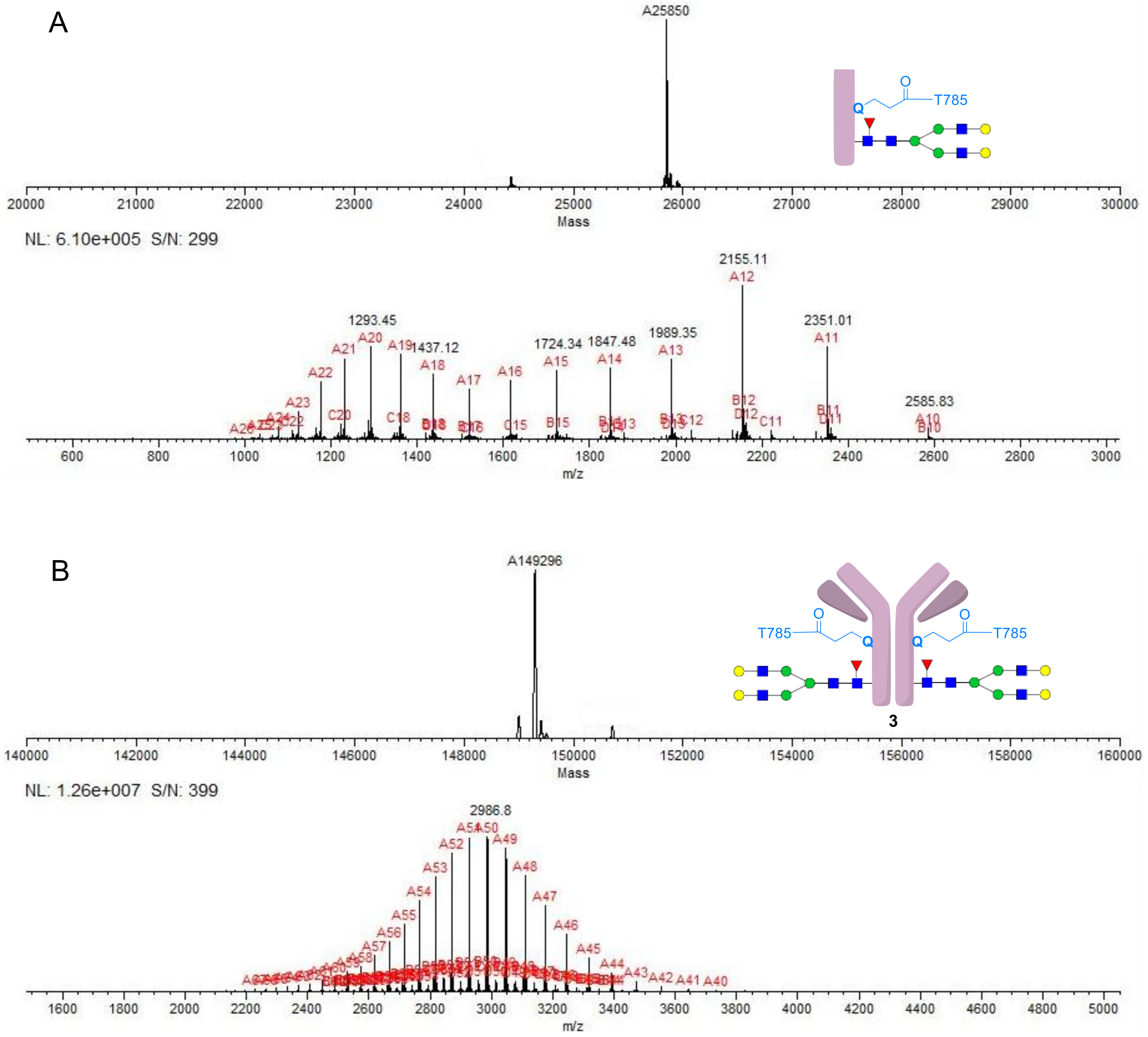


**Fig. S7.** A) Deconvoluted MS spectra of IdeS-digested Fc fragment from **3**. B) Intact-antibody MS spectra of **3**

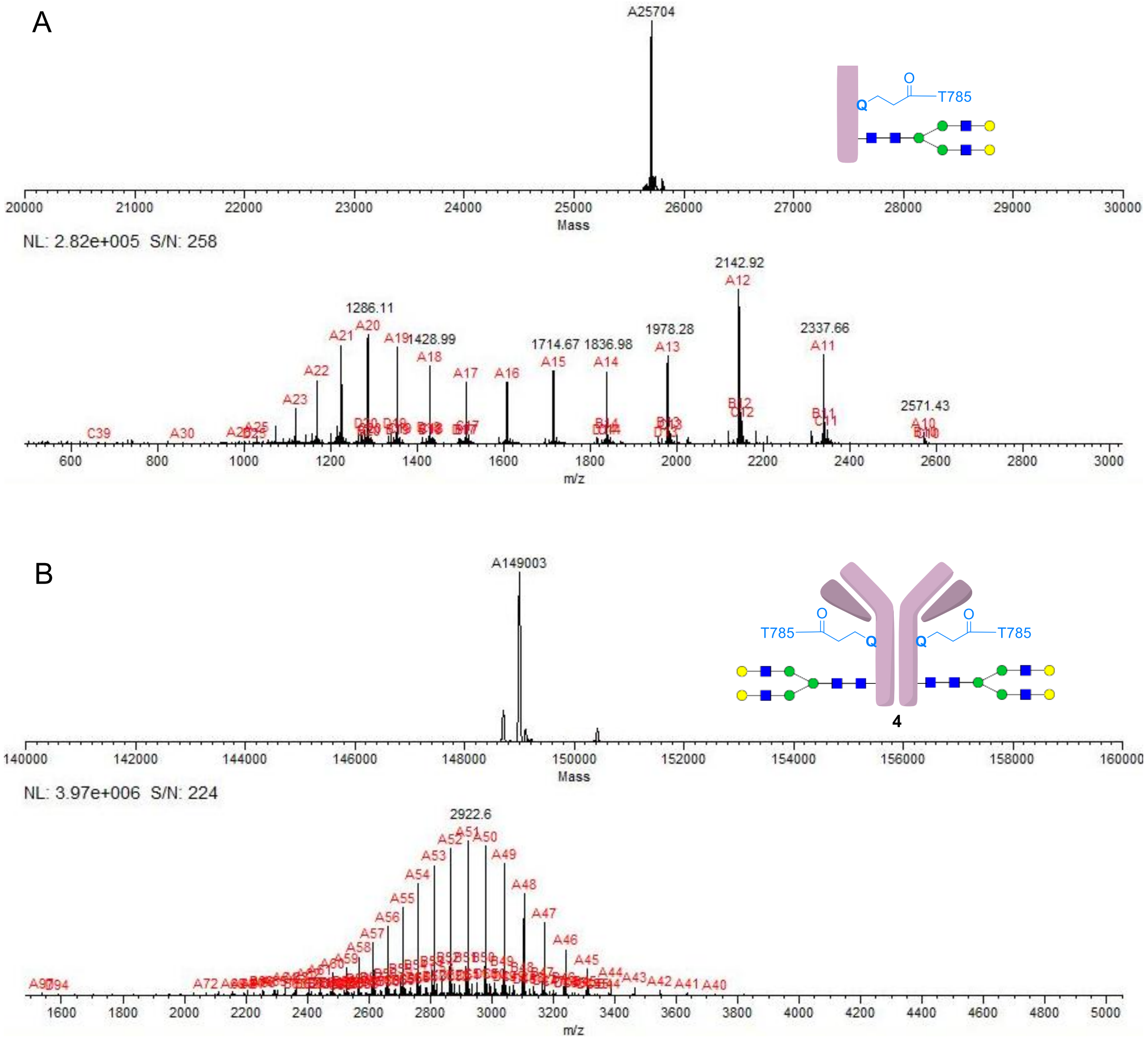


**Fig. S8.** A) Deconvoluted MS spectra of IdeS-digested Fc fragment from **4**. B) Intact-antibody MS spectra of **4**

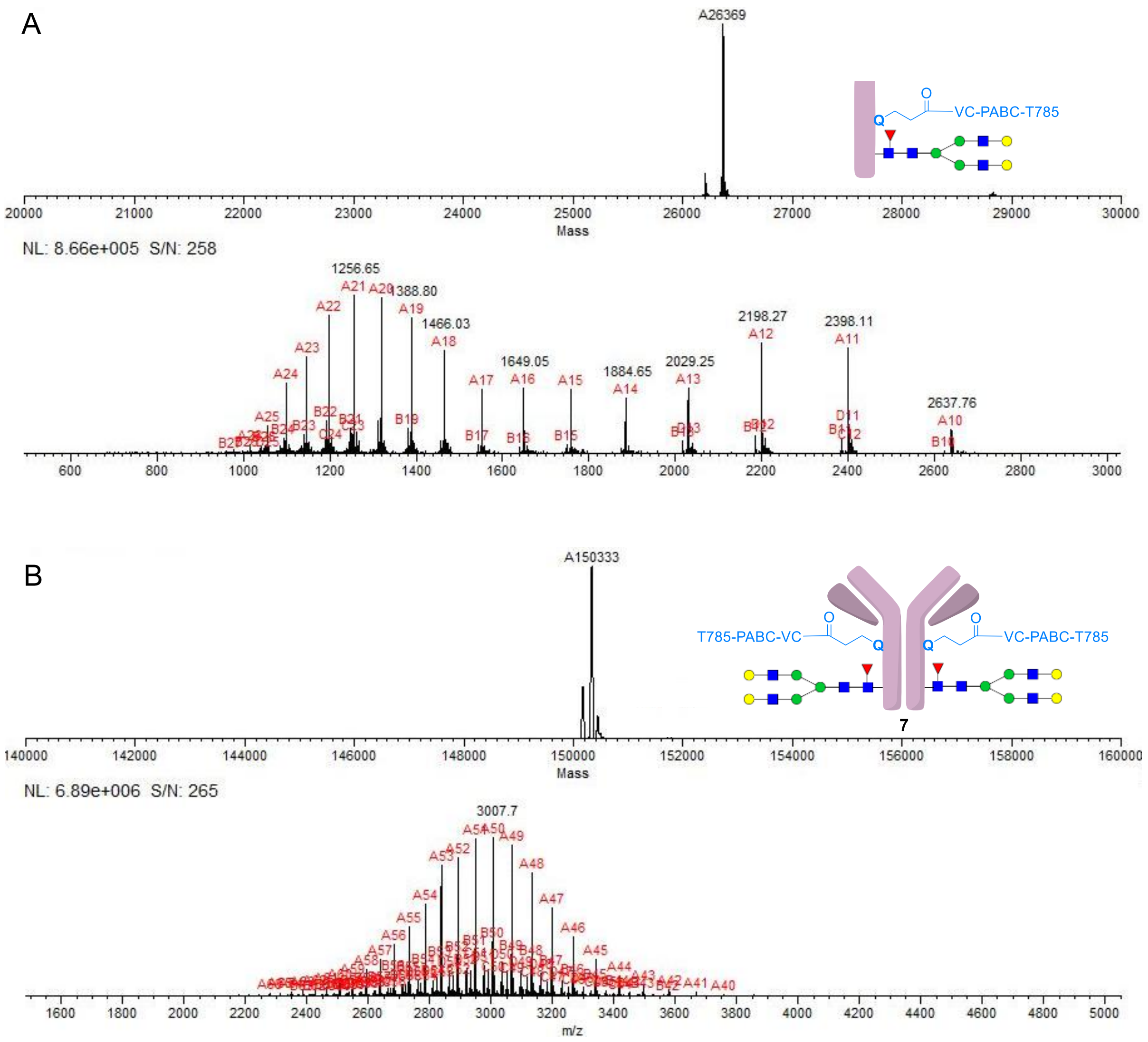


**Fig. S9.** A) Deconvoluted MS spectra of IdeS-digested Fc fragment from **7**. B) Intact-antibody MS spectra of **7**

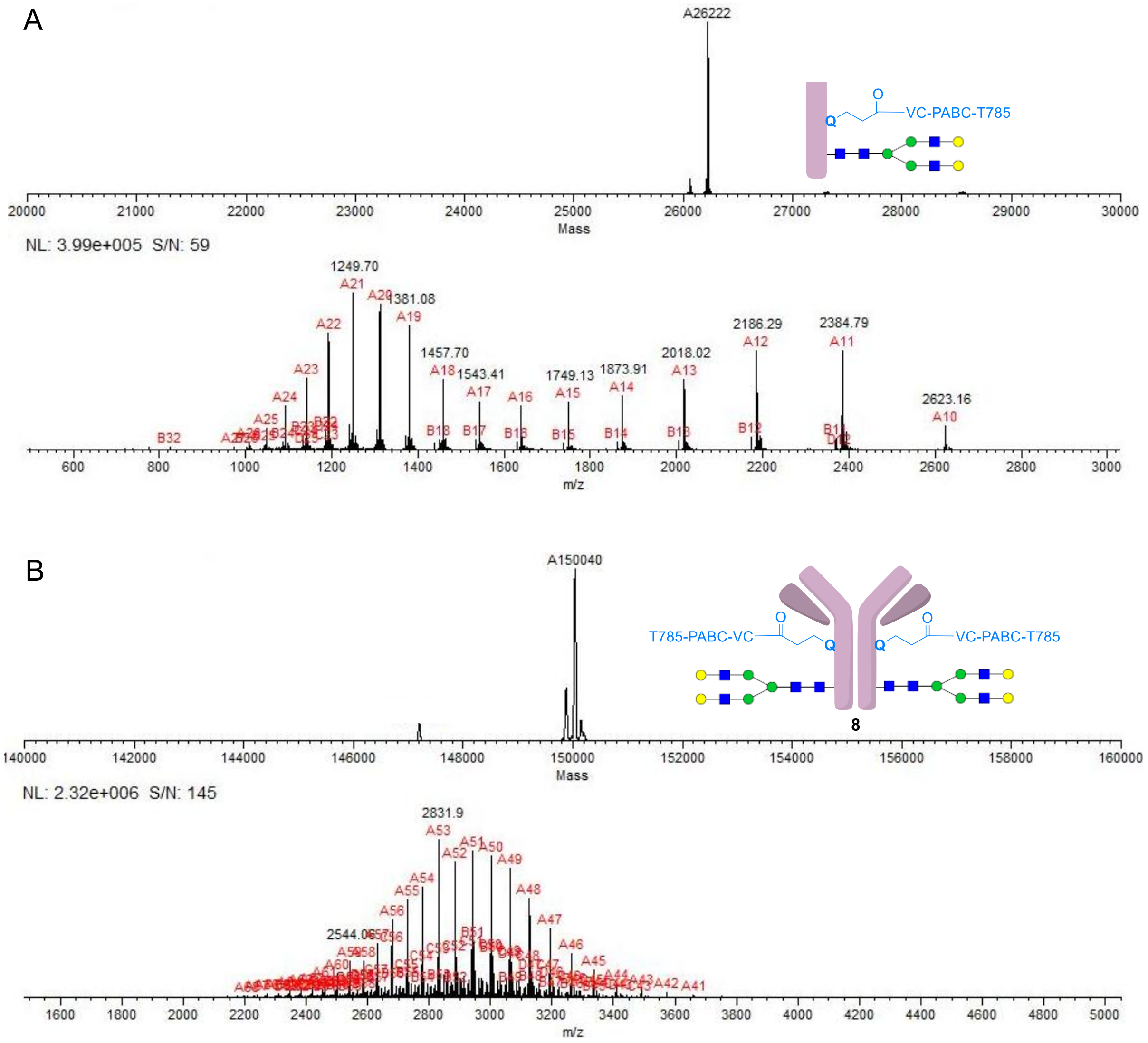


**Fig. S10.** A) Deconvoluted MS spectra of IdeS-digested Fc fragment from **8**. B) Intact-antibody MS spectra of **8**

**SDS-PAGE analysis of ISACs**

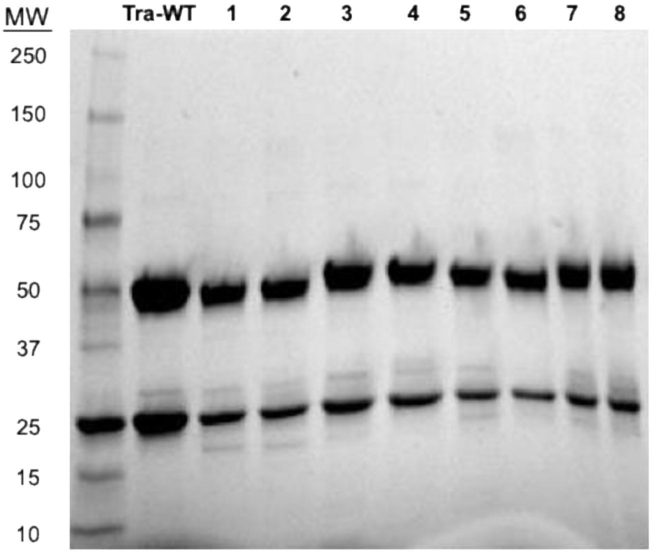


**Fig. S11.** SDS-PAGE analysis of ISACs under denaturing conditions. The ~50 kDa bands correspond to antibody heavy chains, while the ~25 kDa bands correspond to light chains. A clear upward shift of the heavy-chain bands was observed for ISACs **3**, **4**, **7**, and **8** compared with WT and ISACs **1**, **2**, **5**, and **6**, consistent with successful payload conjugation and Fc glycan remodeling.

## HER2-binding analysis of ISACs on SKBR3 cells by flow cytometry

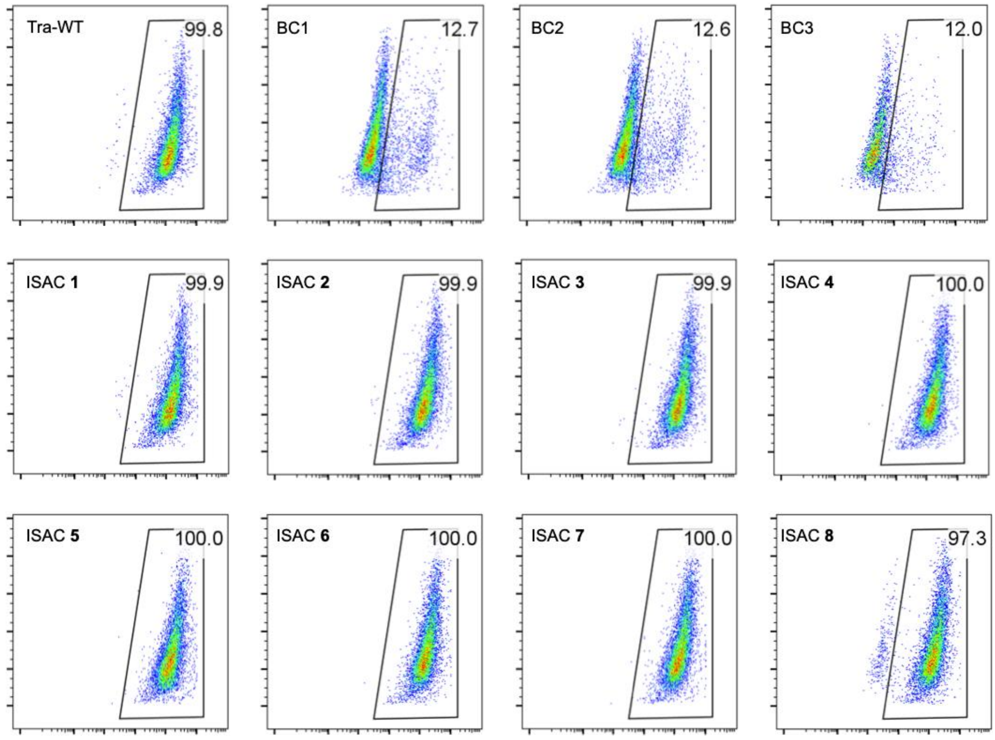


**Fig. S12.** Representative flow cytometry plots of HER2 binding on SKBR3 cells by the ISACs. SKBR3 cells were incubated with Tra-WT or the indicated ISACs at 100 nM, followed by staining with PE-conjugated goat anti-human IgG (H+L) secondary antibody. BC1-BC3 (black control 1-3): Tra-WT only; secondary-antibody only; cell only.

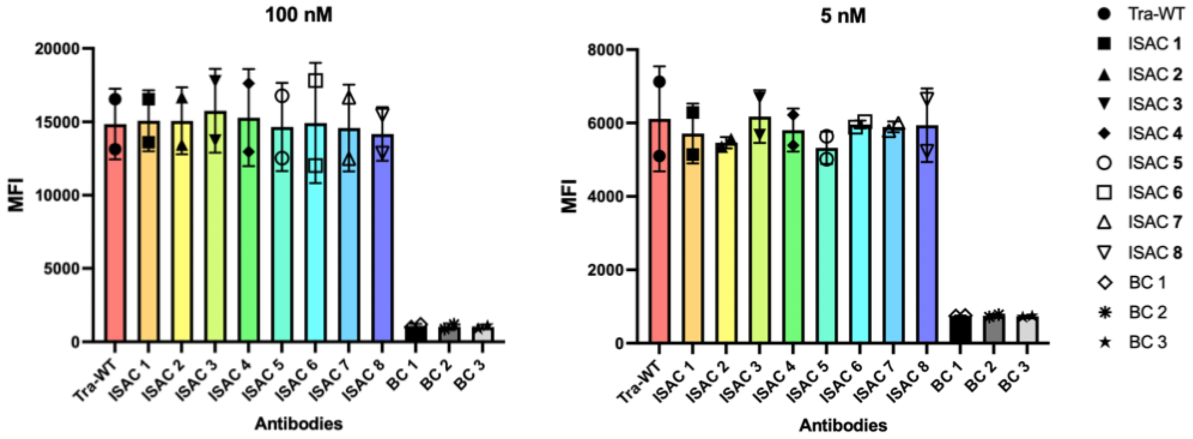


**Fig. S13.** Quantification of HER2 binding on SKBR3 cells by flow cytometry. SKBR3 cells were incubated with trastuzumab or the indicated ISACs at 5 nM or 100 nM, followed by staining with PE-conjugated goat anti-human IgG (H+L) secondary antibody. HER2 binding was quantified by PE mean fluorescence intensity (MFI). Data represent mean ± SD from duplicate wells.
